# MicroRNA-21 is Induced by Rapamycin in a Model of Tuberous Sclerosis (TSC) and Lymphangioleiomyomatosis (LAM)

**DOI:** 10.1371/journal.pone.0060014

**Published:** 2013-03-29

**Authors:** Anil J. Trindade, Douglas A. Medvetz, Nicole A. Neuman, Faina Myachina, Jane Yu, Carmen Priolo, Elizabeth P. Henske

**Affiliations:** Division of Pulmonary and Critical Care Medicine, Brigham and Women's Hospital and Harvard Medical School, Boston, Massachusetts, United States of America; University of Barcelona, Spain

## Abstract

Lymphangioleiomyomatosis (LAM), a multisystem disease of women, is manifest by the proliferation of smooth muscle-like cells in the lung resulting in cystic lung destruction. Women with LAM can also develop renal angiomyolipomas. LAM is caused by mutations in the tuberous sclerosis complex genes (*TSC1* or *TSC2)*, resulting in hyperactive mammalian Target of Rapamycin (mTOR) signaling. The mTOR inhibitor, Rapamycin, stabilizes lung function in LAM and decreases the volume of renal angiomyolipomas, but lung function declines and angiomyolipomas regrow when treatment is discontinued, suggesting that factors induced by mTORC1 inhibition may promote the survival of TSC2-deficient cells. Whether microRNA (miRNA, miR) signaling is involved in the response of LAM to mTORC1 inhibition is unknown. We identified Rapamycin-dependent miRNA in LAM patient angiomyolipoma-derived cells using two separate screens. First, we assayed 132 miRNA of known significance to tumor biology. Using a cut-off of >1.5-fold change, 48 microRNA were Rapamycin-induced, while 4 miRs were downregulated. In a second screen encompassing 946 miRNA, 18 miRs were upregulated by Rapamycin, while eight were downregulated. Dysregulation of miRs 29b, 21, 24, 221, 106a and 199a were common to both platforms and were classified as candidate “RapamiRs.” Validation by qRT-PCR confirmed that these microRNA were increased. miR-21, a pro-survival miR, was the most significantly increased by mTOR-inhibition (p<0.01). The regulation of miR-21 by Rapamycin is cell type independent. mTOR inhibition promotes the processing of the miR-21 transcript (pri-miR-21) to a premature form (pre-miR-21). In conclusion, our findings demonstrate that Rapamycin upregulates multiple miRs, including pro-survival miRs, in TSC2-deficient patient-derived cells. The induction of miRs may contribute to the response of LAM and TSC patients to Rapamycin therapy.

## Introduction

Lymphangioleiomyomatosis (LAM) is a devastating multi-system disease that almost exclusively affects women and can result in end-stage lung disease. LAM is characterized by the diffuse proliferation of smooth muscle-like cells (LAM cells) that express melanocyte lineage proteins. In the lungs, LAM cells can lead to small airway obstruction, blockage of lymphatic vessels leading to the formation of chylous pleural effusions, and cystic parenchymal destruction which is believed to be due to the release of matrix metalloproteinases and other catabolic enzymes [1], [2]. LAM occurs in two forms: in association with germline mutations in the tuberous sclerosis complex (TSC) genes, and in women who do not have tuberous sclerosis (sporadic LAM). The majority of women with TSC-LAM and about 40% of women with sporadic LAM have renal angiomyolipomas, which are benign tumors consisting of smooth muscle, fat and dysplastic vasculature. Angiomyolipoma cells have many similarities to LAM cells and may arise from a common progenitor cell [3]. LAM cells from women with sporadic LAM can carry somatic mutations in the *TSC2* gene, which is a tumor suppressor gene that regulates the mammalian Target of Rapamycin (mTOR) [4], [5], [6], [7], [8].

mTOR is a kinase that integrates cellular and environmental cues, including growth factor activity and glucose levels, to regulate cell growth and proliferation. mTOR exists in two distinct complexes: mTOR complex 1 (mTORC1), which includes Raptor, and mTOR complex 2, which includes Rictor [9], [10]. Recent clinical trials of allosteric mTORC1-inhibitors such as Sirolimus (Rapamycin) in women with LAM have been promising in that they confer a partial reduction in angiomyolipoma volume and stabilization of lung function [11], [12]. However, upon cessation of therapy lung function decline resumes and angiomyolipomas regrow, suggesting that allosteric mTORC1 inhibitors exert a cytostatic but not cytotoxic effect.

MicroRNA (miRNA or miRs) are small RNA molecules that regulate gene expression, primarily by affecting transcript stability. Many miRNA have their own promoter regions and undergo transcription in a tightly regulated manner. Mature miRNA form a RNA-induced silencing complex (RISC) with chaperone proteins, binding to the 3′ untranslated region of mRNA to either promote transcript degradation or repress protein translation. Over 1000 miRNA species have been identified, each with the ability to regulate hundreds of genes; moreover the 3′UTR of individual genes can recognize multiple miRNAs and mRNAs of differing abundance can “compete” for miRNA binding and consequent silencing [13].

Given the increasing evidence that microRNA participate in the pathogenesis of malignancies, inflammatory bowel disease, cardiomyopathies, and lung diseases including asthma, pulmonary hypertension and idiopathic pulmonary fibrosis [14], [15], [16], [17], [18], we explored whether dysregulated miRNA expression is associated with mTORC1 activity in LAM and TSC. We utilized TSC2-deficient LAM patient-derived cells with hyperactive mTORC1 and two different miRNA screens to identify “RapamiRs”- miRNA that are modulated by the mTORC1-inhibitor, Rapamycin. Using qRT-PCR, we confirmed the dysregulation of six miRs by Rapamycin and focused our attention on miR-21, which is a known “oncomiR.” Interestingly, miR-21 induction by Rapamycin appears to be TSC2-independent.

Our results demonstrate that Rapamycin promotes the expression of several miRNA species, which we term “RapamiRs,” in cellular models of LAM. Of the RapamiRs identified in our screens, miR-21, which has pro-survival effects in many cell types, is the most strongly induced by Rapamycin. To our knowledge, this is the first unbiased identification of mTORC1-dependent miRNA in human cells. Manipulation of pro-survival mTORC1-dependent miRNA may prove to be a valuable adjunct to Rapamycin therapy for TSC and LAM patients. RapamiRs may also have relevance to the broad range of human diseases that are associated with activation of mTORC1, including the majority of human malignancies.

## Results

### High-throughput screening identifies Rapamycin-dependent microRNA

**Figure 1:**
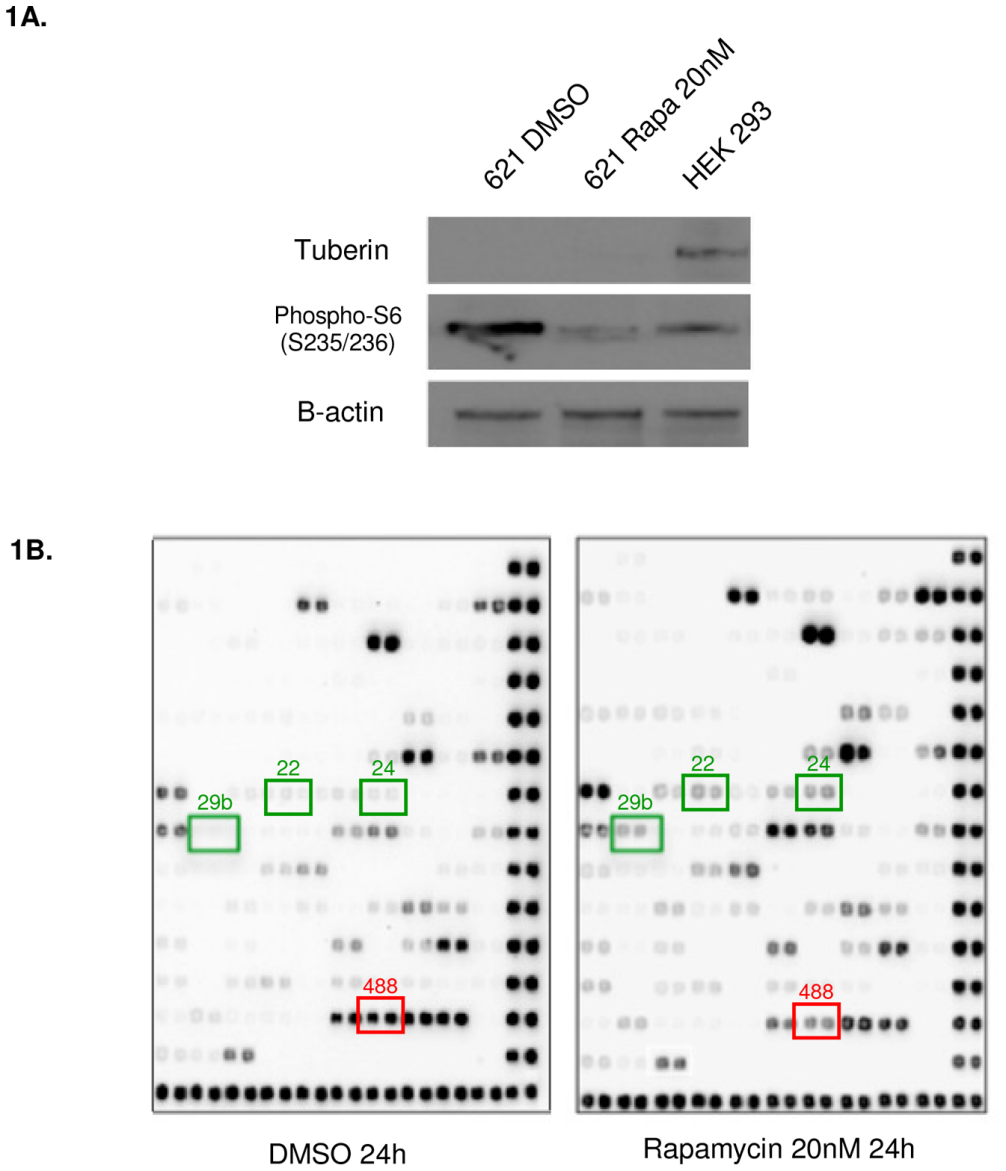
Rapamycin regulates miRNA in TSC2-deficient LAM patient angiomyolipoma-derived cells. **A**) Western blot demonstrating that Rapamycin inhibits phosphorylation of ribosomal protein S6 (S235/236) in TSC2-deficient 621-101 cells. **B**) Signosis miRNA microarray identifies Rapamycin-dependent miRNA; boxed dots display an induction of miR-22, 24, and 29b (in green) and a decrease in miR-488 (in red) amongst others by Rapamycin. The array assays cancer-specific miRNA in duplicate.

To determine whether Rapamycin regulates miRNA, we performed two unbiased miRNA screens using LAM patient-derived 621-101 cells as an *in vitro* model of LAM (Tables 1, 2). These cells were derived from the angiomyolipoma of a woman with the sporadic form of LAM and carry bi-allelic inactivation of the *TSC2* gene [19]. An identical TSC2 mutation (R611Q) was present in this patient's pulmonary LAM cells [5]. 621-101 cells, cultured in DMEM containing 10% FBS, were treated with the allosteric mTOR inhibitor, Rapamycin (20 nM, 24 hours) or with vehicle control (DMSO). Isolated RNA was applied to a Signosis microRNA platform which assays 132 cancer-specific human miRNA by ligating two complementary tagged DNA probes to each target miRNA and hybridizing the complexes to a nitrocellulose membrane. Each target is assayed in duplicate and normalized to the expression of RNU48, a small nucleolar RNA molecule commonly used for normalization of miRNA levels [20]. The western blot in **Figure 1A** shows the level of phospho-S6 downregulation with rapamycin treatment. Nineteen miRNA were induced more than 2-fold, with miRs- 29b, 22, 26a, 199b and 181a being most highly increased. Four miRs - 375, 488, 142-5p, and 368 - were downregulated (**Figure 1B**). These results point toward a previously unknown miR-regulated signaling network downstream of mTORC1 with the potential to impact the expression of hundreds of mRNA in a cell-type dependent manner. We termed these Rapamycin-dependent miRs “RapamiRs.”

**Table 1:** Rapamycin-regulated miRNA in LAM patient-derived cells identified by the Signosis Array (Fold Change >1.5).

miRNA	Fold Change (Rapa 24 h/DMSO	miRNA	Fold Change (Rapa 24 h/DMSO)	miRNA	Fold Change (Rapa 24 h/DMSO)
miR-29b	4.47	miR-206	2.21	miR-10a	1.63
miR-22	2.99	miR-224	1.97	miR-136	1.62
miR-26a	2.95	miR-125a	1.95	miR-182	1.62
miR-199b	2.93	miR-106b	1.92	miR-188	1.62
miR-181a	2.82	miR-154	1.88	miR-221	1.60
miR-30b	2.69	miR-9	1.87	miR-34a	1.59
miR-19a	2.64	miR-21	1.85	miR-181d	1.58
miR-125b	2.52	miR-204	1.85	miR-20a	1.57
miR-137	2.48	miR-9-1	1.83	miR-10b	1.57
miR-181b	2.47	Let-7b	1.80	miR-126	1.57
miR-181c	2.47	miR-140	1.76	miR-30c	1.56
miR-30a-5p	2.47	miR-106a	1.74	miR-131	1.56
miR-17-3p	2.44	miR-30a-3p	1.72	miR-17-5p	1.53
miR-197	2.30	miR-24	1.71	miR-368	0.67
miR-199a	2.30	miR-135b	1.70	miR-142-5p	0.65
miR-183	2.29	miR-141	1.70	miR-488	0.56
miR-18a	2.23	miR-19b	1.70	miR-375	0.54
miR-95	2.21				

**Table 2:** Rapamycin-regulated miRNA in LAM patient-derived cells identified by the Exiqon Array (Fold Change >1.5, normalized to RNU44).

microRNA	Expression Change (Rapamycin/DMSO)	microRNA	Expression Change (Rapamycin/DMSO)
miR-29b	2.65	miR-1274	0.67
miR-31	2.64	miR-210	0.67
miR-29a	2.15	miR-451	0.64
miR-222	2.14	miR-1285	0.62
miR-300	2.13	miR-1308	0.62
miR-21	2.07	miR-1908	0.58
miR-1973	1.97	miR-513a-5p	0.43
miR-221	1.91	miR-1275	0.42
miR-886-5p	1.86		
miR-24	1.82		
miR-574	1.73		
miR-106a	1.62		
let-7d	1.57		
mir-886-3p	1.55		
miR-23b	1.53		
miR-214	1.53		

**Figure 2:**
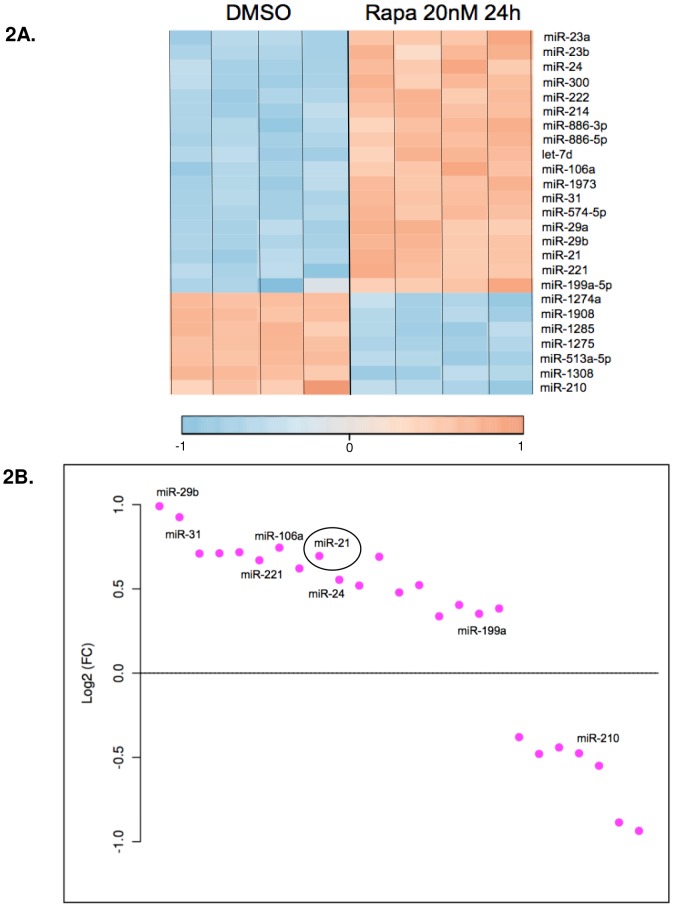
Exiqon miRNA microarray confirms 8 Rapamycin-dependent miRNA. 621-101 cells were treated with Rapamycin 20 nM or DMSO for 24 hours. Total RNA was isolated and applied to the Exiqon platform, which assays 946 human miRNA. **A**) Heat map of miRNA dysregulated by Rapamycin >1.5-fold, log_2_ scale. RNA from three biologic replicates per condition was pooled; each miRNA was assayed in quadruplet on the array. **B**) miRNA dysregulated by Rapamycin >1.5-fold (normalized to RNU44). Highlighted miRNA (except miR-31 and 210) are common to both the Exiqon and Signosis platforms. miR-21 is circled.

To further investigate this putative RapamiR network, we performed a second, broader screen using the Exiqon platform, which incorporates locked-nucleic acid technology to increase probe-target specificity. This platform assays 946 human miRNA in quadruplet, including all known human miRNA listed in miRBase (version 15.0, http://mirbase.org/). A pool of three biological replicates was used for each sample. Results were normalized to RNU44. Interestingly, only 18 miRNAs were upregulated >1.5-fold (**Figure 2**), versus 19 miRNA upregulated >2 fold and 48 upregulated >1.5 fold in the Signosis array (Figure 1), despite the fact that the Signosis array contained fewer miRNA. miRs 29b, 31, 29a, 222 and 300 were among the most strongly induced miRNA using the Exiqon platform; 8 miRs were downregulated >1.5 fold.

Upregulation of miRNA 29b, 21, 24, 221, 106a and 199a was common to both the Signosis and Exiqon platforms. Of the miRs assayed in the Exiqon array that were not included in the Signosis array, Rapamycin promoted a >1.5-fold induction of miR-31, 222, 300, 1973, 886-5p, 886-3p, 23 and 214 and a >1.5-fold repression of miRs 210, 451, 513 and 1275.

**Figure 3:**
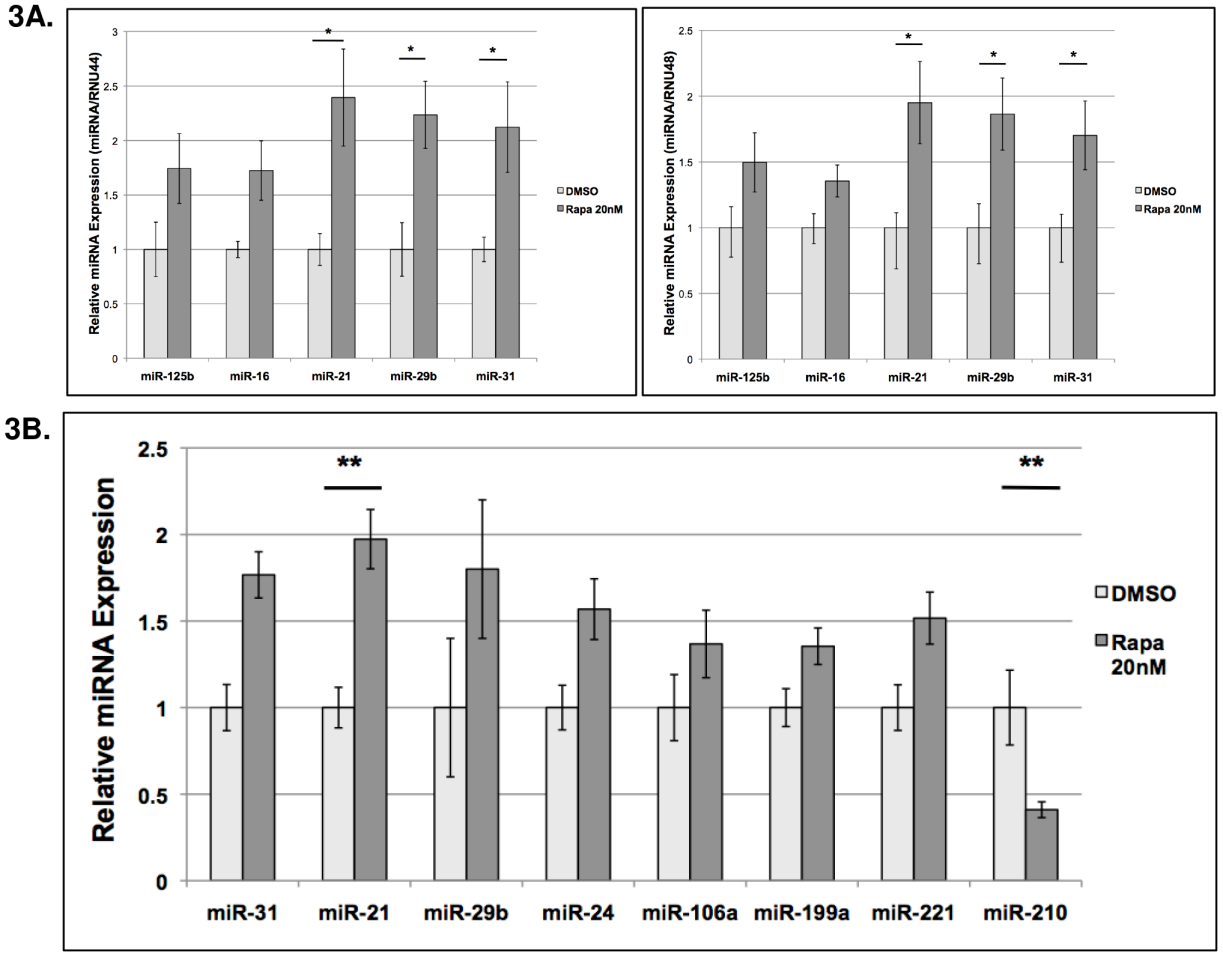
qRT-PCR confirmation of Rapamycin-dependent miRNA in TSC2-deficient cells. TSC2−/− cells were treated with Rapamycin 20 nM or DMSO for 24 hr and miRNA expression was assessed by qRT-PCR. **A**) miRNA expression is similar in 621-101 cells using RNU44 (left panel) or RNU48 (right panel) for normalization. **B**) miRNA expression in 621-101 cells normalized to RNU44. Highlighted results are significant using a Bonferroni correction.

The miRs that were dysregulated in both screens (miRs- 29b, 21, 24, 221, 106a and 199a) were included in a set selected for qRT-PCR confirmation. We also included miR-31 and miR-210, which were among the twelve miRNA dysregulated in the Exiqon array but not included in the Signosis array. miR-31, which was upregulated by Rapamycin, is anti-tumorigenic, preventing metastasis in models of breast cancer [21], [22], and miR-210, which was downregulated by Rapamycin, is a well-described hypoximiR [23], [24], [25]. HypoximiRs are upregulated during hypoxia and have been shown to promote angiogenesis and smooth muscle proliferation. We first confirmed that similar levels of RapamiRs were observed using either RNU44 or RNU48 to normalize miRNA expression (**Figure 3A**). Three different biological replicates were used for the confirmation studies and miRNA expression was normalized to RNU48. MiRNA 21, 24, 31 and 221 were induced by Rapamycin >1.5-fold, consistent with the array results, with miR-21 being the most highly induced (**Figure 3B**). MiR-210 was decreased >1.5-fold, again consistent with the array results (Figure 3B).

### Rapamycin induces miR-21 expression in multiple TSC2-deficient cell types and miR-21 expression may be TSC2-independent

**Figure 4:**
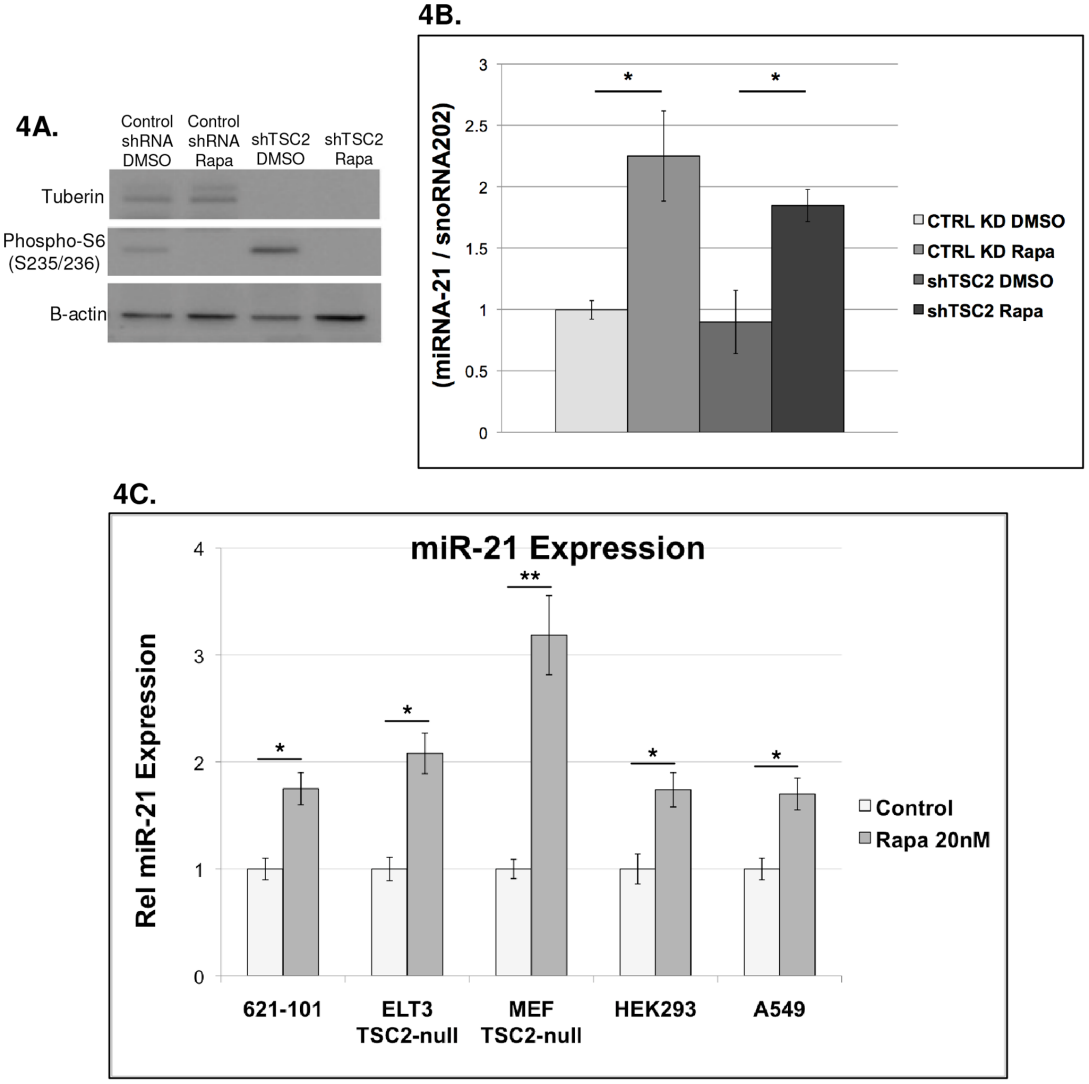
miR-21 is mTOR-dependent and may be TSC2-independent. **A**) Stable downregulation of tuberin in C3H-10T1/2 pre-pericytes results in increased phosphorylation of ribosomal protein S6, as expected. Treatment with Rapamycin (20 nM, 24 h) inhibits phosphorylation of S6. **B**) Downregulation of TSC2 in C3H-10T1/2 cells does not affect miR-21 expression. Inhibition of mTORC1 with Rapamycin induces ∼2-fold increase in miR-21 expression in both control shRNA and TSC2 shRNA cells. Bars represent the mean of two biologic replicates +/− SD. * p<0.05. **C**) LAM patient-derived cells (621-101), TSC2-null rat uterine leiomyoma-derived cells (ELT3), TSC2-null mouse embryonic fibroblasts (MEFs), HEK293 and lung adenocarcinoma (A549) cells were treated with Rapamycin 20 nM vs Control for 24 h. Relative MiR-21 expression was determined by qRT-PCR. Human cells were normalized to RNU44, mouse cells to snora202 and rat cells to U87, which are all small nucleolar RNA molecules. For all charts, bars represent the mean of three biologic replicates +/− standard error. * p<0.05. ** p<0.01.

To identify whether miR-21 is regulated by Rapamycin and/or TSC2 in other cell lines, tuberin (TSC2) was stably downregulated in C3H-10T1/2 mouse pre-pericyte fibroblasts with a lentiviral shRNA vector (**Figure 4A**). Cells were cultured in DMEM containing 10% FBS and treated with Rapamycin (20 nM) or control for 24 hours. Rapamycin increased miR-21 levels approximately 2-fold in both tuberin-deficient and control shRNA cells (**Figure 4B**), but unexpectedly no decrease in miR-21 was observed in the cells with tuberin downregulation despite the increased phosphorylation of ribosomal protein S6, a downstream target of mTORC1. Next, we utilized Tsc2-null mouse embryonic fibroblasts and ELT3 cells (Tsc2-null cells from an Eker rat uterine leiomyoma), which are established cellular models of TSC [26], [27], [28] compared to HEK293 and A549 cells, which express TSC2. Cells were cultured in DMEM containing 10% FBS and treated with Rapamycin (20 nM, 24 h) versus vehicle control. The human cells were normalized to RNU44, murine cells to snoRNA202, and rat cells to U87. miR-21 was induced >1.5-fold (p<0.05, n = 3) by Rapamycin in each of these cell lines (**Figure 4C**). These results further confirm that miR-21 is induced by Rapamycin in different cell lineages and species and suggest that the regulation of miR-21 may be TSC2-independent.

### Rapamycin regulates miR-21 levels independently of AKT signaling

**Figure 5:**
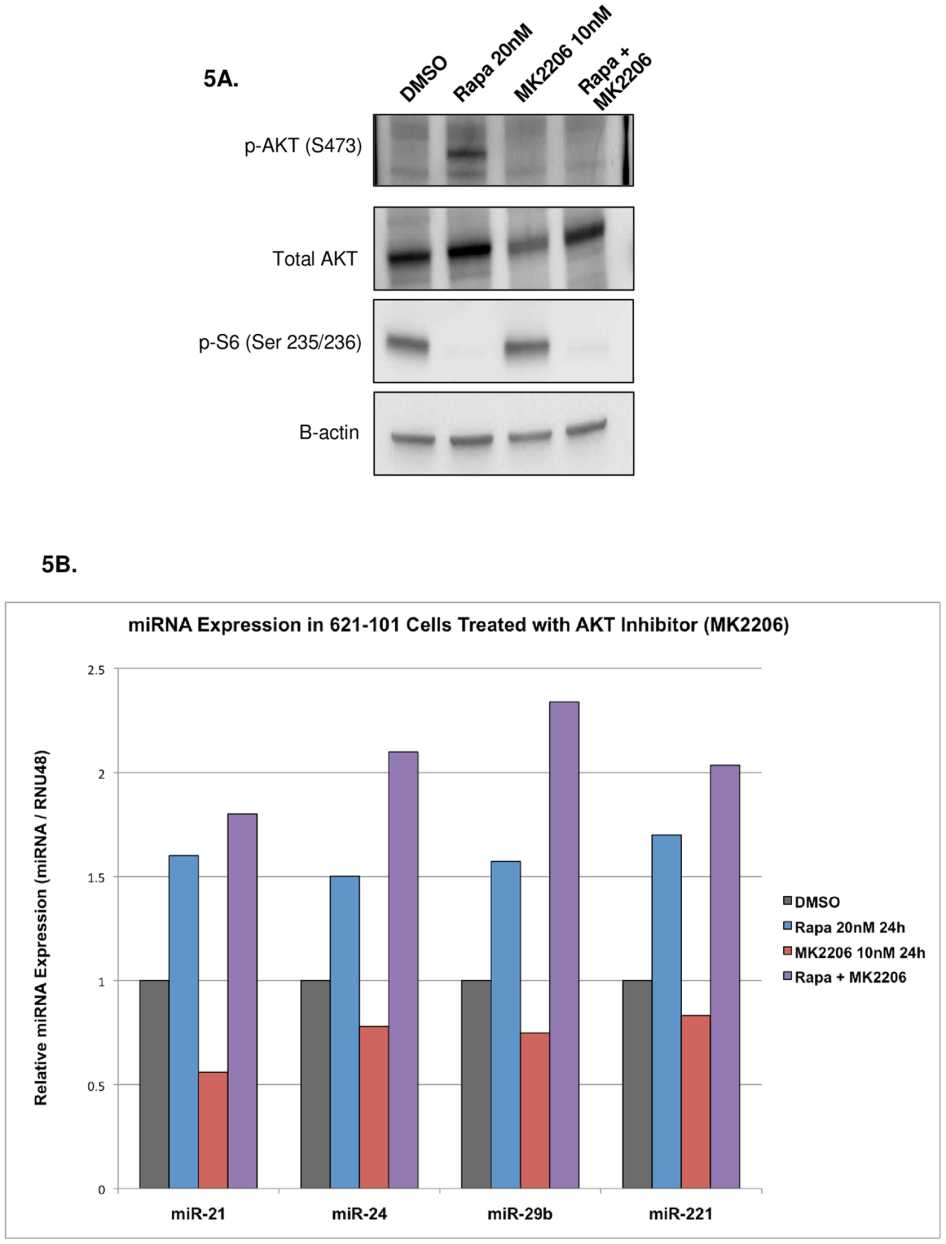
Rapamycin induces miR-21 expression via an AKT-independent mechanism. **A**) Western blot analysis of 621-101 cells treated with DMSO (lane 1), Rapamycin (20 nM, 24 h - lane 2), the AKT inhibitor MK2206 (10 nM, 24 h - lane 3), and Rapamycin and MK2206 (lane 4). Rapamycin treatment induces AKT phosphorylation at S473 and MK2206 abrogates Rapamycin's effect on phosphor-Akt. **B**) Expression of miR-21, 24, 29b, and 221 in 621-101 cells treated as in A). miR-21 levels are induced by Rapamycin, however the addition of MK2206 has no effect suggesting an AKT-independent mechanism.

TSC2-null cells display feedback inhibition to the PI3K/AKT signaling pathway via hyperactivation of mTORC1 [29], [30]. When TSC2-null cells are treated with Rapamycin this feedback inhibition is released and AKT is phosphorylated and activated. To determine whether Rapamycin regulates miR-21 expression via an AKT-dependent mechanism, we treated 621-101 cells with Rapamycin and the AKT inhibitor MK2206 (Selleckchem, Catalog No. S1078) (**Figure 5**). In Figure 5A (lanes 1–2), western blot analysis was performed to confirm that p-AKT levels are elevated in 621-101 cells treated with Rapamycin (20 nM, 24 h) compared to DMSO treated cells. Additionally, we confirmed that MK2206 (10 nM, 24 h) inhibited AKT phosphorylation at S473 when 621-101 cells were treated with Rapamycin and MK2206. In Figure 5B, we analyzed miR-21 levels in the same 621-101 cell samples used for western blot. miR-21 levels were upregulated by Rapamycin and unaffected by AKT inhibition (compare blue bar to purple bar). These results indicate that miR-21's regulation by Rapamycin is AKT-independent in 621-101 cells.

### Rapamycin potentiates the post-transcriptional processing of pri-miR-21

**Figure 6:**
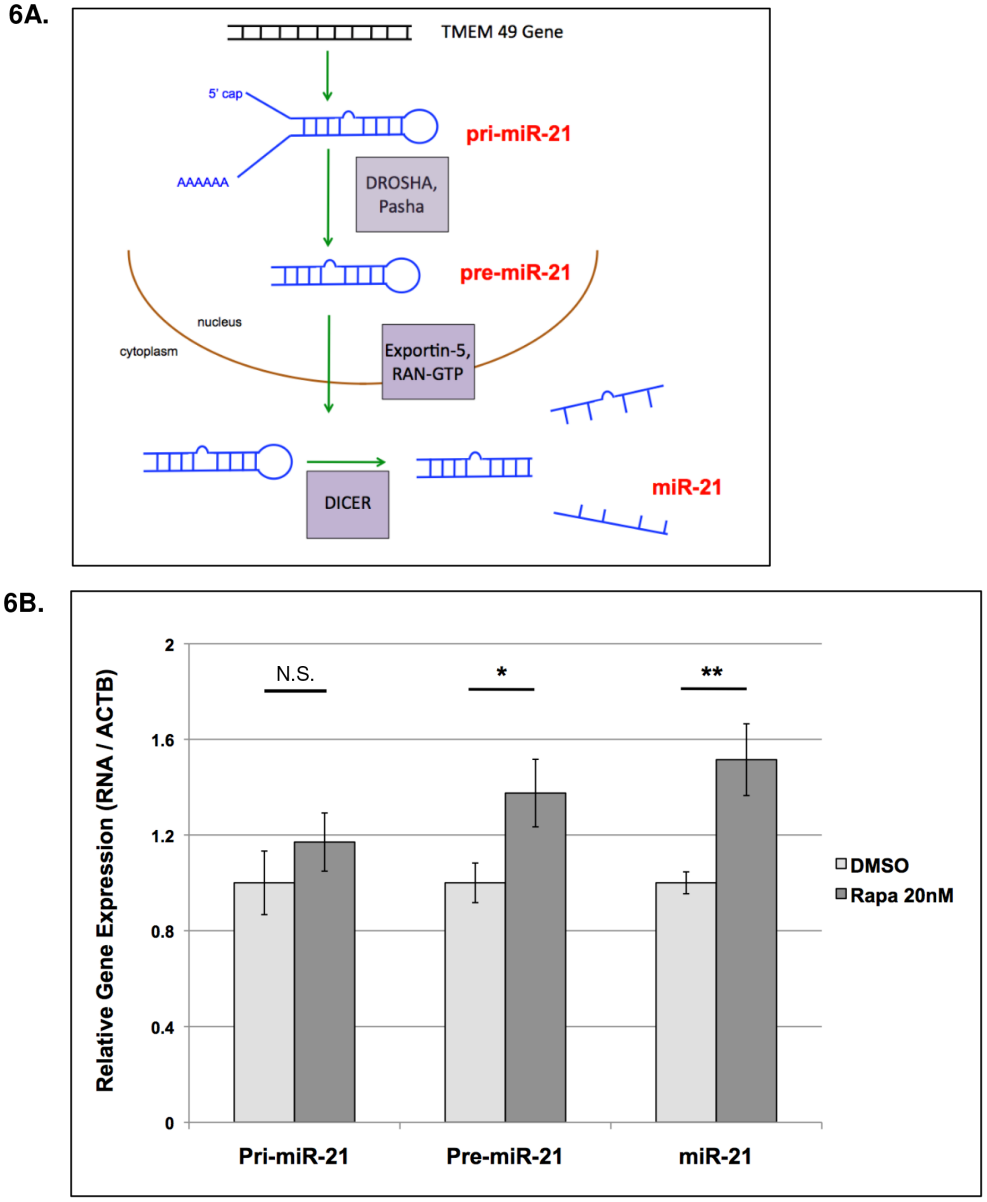
Rapamycin promotes the post-transcriptional processing of pre-miR-21. **A**) The biogenesis of mature miR-21. Pri-miR is transcribed from the intronic region of the TMEM49 gene and processed into pre-miR-21 by the Drosha/Pasha complex and further processed by Dicer to create the mature form. **B**) 621-101 cells were treated with Rapamycin 20 nM or control (DMSO) for 24 hours. Total RNA was extracted and assayed by qRT-PCR for the differential expression of pri-miR-21, pre-miR-21 and miR-21. Expression of pre-miR-21 was significantly induced by Rapamycin, whereas pri-miR-21 levels were not affected, suggesting that Rapamycin promotes the Drosha-mediated post-transcriptional processing of pri-miR-21 into pre-miR-21 at 24 hours. Bars represent the mean of three biological replicates +/− SD. * p<0.05, ** p<0.01.

MiRNA biogenesis is a tightly regulated process, with key enzyme complexes participating in transcription and processing at three major junctions (**Figure 6A**). Post-transcriptional processing is a key mechanism to controlling miRNA levels [31]. The regulation of miR-21, in particular, has been well-characterized, with Davis and colleagues demonstrating that TGF-beta and Smad signaling induces a Drosha-mediated post-transcriptional processing of pri-miR-21 to induce miR-21 expression [32]. To determine whether Rapamycin influences the processing of pri-miR-21, we treated 621-101 cells, cultured in DMEM containing 10% FBS, with Rapamycin (20 nM) or DMSO for 24 hours and assayed the expression of pri-miR-21, pre-miR-21 and miR-21 using qRT-PCR. Rapamycin significantly induced pre-miR-21 and miR-21 at 24-hours, but did not affect expression of pri-miR-21 (**Figure 6B**). This result suggests that Rapamycin potentiates the DROSHA-mediated processing of pri-miR-21.

## Discussion

The landmark Multicenter International LAM Efficacy of Sirolimus (MILES) Trial, a randomized, placebo controlled trial in 89 women with sporadic or TSC-associated LAM, demonstrated that Rapamycin (an allosteric mTOR inhibitor) stabilized lung function during one year of therapy; discontinuation of therapy resulted in a rate of lung function decline similar to untreated patients [12]. These findings are consistent with results from an earlier phase I/II trial of patients with angiomyolipomas, some of who also had sporadic or TSC-associated LAM, in which Rapamycin promoted a decrease in the volume of renal angiomyolipomas by almost 50%, with regrowth to approximately the original size after treatment was stopped [11]. These studies suggest that temporarily slows disease progression, but does not eradicate the TSC2-deficient LAM and angiomyolipoma cells. Therapeutic targeting of factors that promote the survival of LAM cells during Rapamycin therapy could lead to more robust and/or durable responses.

While miRNA have been studied in many human diseases, whether miRNA contribute to the therapeutic response to Rapamycin in LAM and TSC is unknown. To address this, we performed two screens to determine whether Rapamycin impacts miRNA levels in LAM patient angiomyolipoma-derived cells. The first screen assayed 132 miRNA of known importance to cancer signaling pathways, while the second was a more comprehensive screen that assayed all 946 known miRNA listed in miRBase Version 15.0. The screens revealed a complex and previously unrecognized network of Rapamycin-regulated miRNA, which we termed “RapamiRs”. Using qRT-PCR to confirm our results, we demonstrate that miR-21 is significantly induced by Rapamycin, whereas miR-210 is repressed. We further analyzed Rapamycin's effect on miR-21, demonstrating that miR-21 is induced in several different TSC2-deficient cell lines. Utilizing mouse pre-pericyte fibroblasts with stable downregulation of tuberin, we confirmed that the dysregulated expression of miR-21 is mTOR-dependent, but tuberin independent. Finally, we revealed that the induction of miR-21 expression by Rapamycin is mediated by post-transcriptional processing of the primary miR-21 transcript, as opposed to increased transcription. These studies reveal a novel regulatory network that is upregulated by Rapamycin.

We hypothesize that the upregulation of miR-21 by Rapamycin in LAM patient-derived cells impacts the therapeutic response to Rapamycin. First, miR-21 is a known “oncomiR,” inhibiting multiple tumor suppressor genes, including phosphatase and tensin homolog (PTEN), programmed cell death 4 (PDCD4) and sprouty 2 (SPRY2) to promote growth, differentiation and proliferation [33], [34], [35], [36], [37]. These pro-survival effects of miR-21 may partially explain why there is a resumption of disease upon treatment discontinuation in LAM patients treated with Rapamycin. Interestingly SPRY2 has previously been shown to regulate mTORC1 signal transduction and vascularization of the lung [38]. Second, miR-21 is a known regulator of smooth muscle morpholology, promoting a de-differentiated state marked by growth and migration, which is essential for angiogenesis [39], [40]. Krymskaya and colleagues have previously demonstrated a role for regulators of smooth muscle function in the pathogenesis of LAM, identifying that RhoA is activated in TSC2-deficient cells [41], [42]. Smooth muscle-like LAM cells can be identified *in vivo* in two different morphologic states, a highly proliferative “spindle shape” and a more static “epithelioid” state [43]; the pathways that regulate the phenotypic switch of LAM cells is unknown. Third, it is possible that miR-21 induces a pro-inflammatory state that promotes the survival and metastasis of TSC2-deficient LAM cells. miR-21 is regulated by inflammatory mediators and in turn acts to promote inflammation. For example, interleukin-6 is a potent modulator of miR-21, acting via STAT3 signaling [44]. Additionally, mTORC1 has been shown to activate STAT3 signaling in mice and humans making this an intriguing link [45], [46]. Moreover, miR-21 inhibits PTEN and PDCD4 to repress NF-kB signaling and IL-10, an anti-inflammatory interleukin [47]. A number of groups have identified a link between innate immunity pathways and TSC-mTOR signaling. Weichhart and colleagues have shown that TSC2-deficient cells exhibit an anti-inflammatory state via the induction of IL-10, whereas Rapamycin treatment promotes a pro-inflammatory condition by suppressing IL-10 and inducing TNF-alpha, IL-6, and IL-12p40 [48]. Moss and colleagues have also contributed to the recognition of inflammatory processes underlying the pathogenesis of LAM, demonstrating that chemokines and chemokine receptors, especially CCL-2/MCP-1, are dysregulated in LAM cells and in bronchoalveolar lavage fluid obtained from LAM patients [49]. Therefore, the induction of miR-21 by Rapamycin may induce a proliferative smooth muscle morphology and contribute to a pro-inflammatory milieu.

Our identification of Rapamycin-induced miRNA reveals a novel and complex signaling network downstream of mTOR with potential therapeutic implications for women with LAM and patients with TSC receiving Rapamycin therapy. For example, if miR-21 induction by Rapamycin proves to be a strong pro-survival stimulus in TSC2-deficient cells cells, then suppression of miR-21 in conjunction with Rapamycin could represent an effective therapeutic strategy for TSC and LAM. Manipulation of miRNA expression is currently being studied as a therapy for malignancies and cardiomyopathies, with promising results in multiple pre-clinical models of disease [50], [51]; ‘antagomiRs’ that suppress miRNA expression are now being tested in clinical trials. RapamiRs may also be useful as serum biomarkers of response to Rapamycin. Finally, our findings may be relevant to other diseases in which Rapamycin and its analogs are currently being used therapeutically, including cancer, for which there are currently more than 100 active cancer clinical trials using mTOR inhibitors.

## Methods

### Cell culture and Tsc2-downregulation

621-101 (The Rothberg Institute), Tsc2-null mouse embryonic fibroblasts (provided by Dr. David Kwiatkowski), HEK293, C3H10T1/2 cells (American Type Culture Collection) and ELT3 cells (provided by Dr. Cheryl Walker) were maintained in DMEM supplemented with 10% FBS (Sigma), Penicillin (50 units/mL) and Streptomycin (50 mg/mL); media for ELT3 cells also contained G418 (0.5 mg/mL). Lentiviral particles expressing shTSC2 were generated by transfecting HEK293T cells with plasmid DNA expressing shTSC2 (or empty vector) and lentiviral packaging particles (VSVG, PLP1, PLP2). Supernatant containing virions was applied to C3H10T1/2 cells (passage 7) for infection. C3H10T1/2 with lentiviral transfection were maintained in DMEM supplemented with 10% FBS (Sigma) with Puromycin (for selection).

### Immunoblot analyses

Lysates were mixed with Laemmli Sample buffer and boiled for 10 minutes. 30 µg of sample were resolved in a 4–12% acrylamide gel and transferred to nitrocellulose membranes (Bio-Rad, Hercules, CA). The membranes were blotted overnight with rabbit anti–phospho-S235/236 S6 ribosomal protein (Cell Signaling), mouse anti-Actin (Sigma-Aldrich), or rabbit anti-Tuberin (Santa Cruz Biotechnology Inc.).

### MicroRNA Screens

621-101 cells were grown in DMEM supplemented with 10% FBS until 60% confluent. Cells were then treated for 24 hours with Rapamycin (20 nM) or equal volume Dimethylsulfoxide (DMSO). Cells were washed twice with phosphate buffered saline and RNA extracted using the miRCURY RNA Isolation Kit (Exiqon). Assaying of microRNA was performed on the Signosis Cancer MicroRNA Array platform and the miRCURY LNA 5^th^ generation microRNA microarray platform (Exiqon), which contained capture probes for all miRNAs annotated in miRBase (version 15.0; http://www.mirbase.org/).

### Real-time quantitative Reverse Transcription - Polymerase Chain Reaction Analysis (qRT-PCR)

Total RNA was extracted using the miRCURY RNA Isolation Kit (Exiqon). For miRNA quantification RNA was reverse-transcribed using a Taq- ManTM microRNA reverse transcription kit, and subjected to real-time PCR using TaqManTM microRNA assay kits (Applied Biosystems). Reactions were performed in triplicate, using an Applied Biosystems Step One Plus instrument. MiRNA expression was normalized to small nuclear RNA (snRNA) RNU44 or RNU48. For quantification of pri-miR-21 and pre-miR-21, total RNA was reverse transcribed and Quantitative PCR was performed using a SYBR-green kit (Applied Biosystems). The following primers were used to assay pre-miR-21: 5′- TGTCGGGTAGCTTATCAGAC-3′ (forward), 5′- TGTCAGACAGCCCATCGACT-3′ (reverse). The following primers were used to assay pre-miR-21: 5′- TTTTGTTTTGCTTGGGAGGA-3′ (forward), 5′- AGCAGACAGTCAGGCAGGAT-3′ (reverse). Assays were performed using three biologic replicates and three technical replicates for each treatment condition. Assays were performed on the Applied Biosystems Step One Plus instrument. Only one PCR product was observed for each assay.
